# Treatment of type 2 diabetes with saxagliptin: a pharmacoeconomic evaluation in Argentina

**DOI:** 10.1186/2191-1991-3-11

**Published:** 2013-04-27

**Authors:** Jorge F Elgart, Joaquin E Caporale, Lorena Gonzalez, Eleonora Aiello, Maximiliano Waschbusch, Juan J Gagliardino

**Affiliations:** 1 CENEXA – Center of Experimental and Applied Endocrinology (UNLP-CONICET La Plata), PAHO/WHO Collaborating Center for Diabetes, La Plata, Argentina; 2 Bristol-Myers Squibb, Buenos Aires, Argentina

**Keywords:** Type 2 Diabetes treatment, Saxagliptin, DPP-4 inhibitors, Pharmacoeconomics, Cost-effectiveness analysis, Latin America, Argentina

## Abstract

**Background:**

The increasing prevalence of diabetes and its inadequate management results in a heavy burden of the disease for the patients, the health and the productive system and the overall community. Consequently, it is necessary to have new effective drugs to treat people with diabetes to decrease such burden. DPP-4 inhibitors can help to cope with this demand, but its usage is challenged by its apparent high cost. The aim of the current study was to compare a simulated cost-effectiveness ratio of metformin (MET) plus one drug of the DPP-4 inhibitors family, saxagliptin (SAXA) or sulfonylurea (SU) treatment during a 20-year period, from the perspective of the social security system, in a cohort of people with Type 2 diabetes (T2DM) who did not attain glycosylated hemoglobin treatment target values only with MET.

**Methods:**

A discrete event simulation model (Cardiff diabetes model) based on UKPDS 68 was used to simulate disease progression and to estimate the economic and health treatment consequences in people with T2DM. The clinical efficacy parameters for SAXA administration were obtained from the literature; local standard costs were considered for drug acquisition, adverse events (AEs), and micro/macrovascular complications. Costs were expressed in US dollars (2009) with an annual 3.5% discount and a 20-year time horizon.

**Results:**

The SAXA + MET treated group had a lower number of non-fatal events than the SU + MET treated group. The model also predicted a lower number of fatal macrovascular events for the SAXA + MET group (149.6 *vs.* 152.8). The total cost of the SAXA + MET cohort was 15% higher than that of the SU + MET cohort. Treatment with SAXA + MET resulted in a higher number of quality-adjusted life years (QALYs) (9.54 *vs.* 9.32) and life-years gained (LYGs) (20.84 *vs.* 20.76) compared to those treated with SU + MET. The incremental cost per QALY and LYG gained was $7,374 and $20,490, respectively.

**Conclusions:**

According to the criteria proposed by the Commission on Macroeconomics and Health, the use of the combination SAXA + MET is highly cost-effective in Argentina.

## Background

The prevalence of diabetes is growing continuously worldwide and in Argentina this prevalence rose from 8.4% in 2005 to 9.6% in 2009 [1,2]. Although tight glycemic control has been shown to decrease significantly the development and progression of diabetes-related complications with the consequent decrease in costs of treatment [3], such control remains elusive for many people with type 2 diabetes (T2DM). In fact, the National Health and Nutrition Examination Survey 2003–2004 found that only 57.1% of patients with diabetes had a glycosylated hemoglobin (HbA_1c_) level below the current treatment target of 7.0% [4]. A comparable situation has been recorded in Argentina [5,6]. Consequently, the availability of effective drugs to attain treatment target values became an urgent demand that triggered the continuous development of new products by the pharmaceutical industry.

In an attempt to increase the percentage of people with HbA_1c_ value at target recommendations, antihyperglycemic drug therapy has become more aggressive in recent years: initial treatment prescription of metformin (MET) associated to lifestyle changes is currently recommended by most international guidelines [7]. Additionally, the available evidence that combinations of different classes of oral agents are more effective to lower glucose than maximal doses of a single drug, lead to recommend early or even initial prescription of combined therapies for the treatment of people with T2DM [8-12]. Although usually effective, this prescriptive attitude increases markedly treatment costs. Additionally, newer classes of drugs such as those of the incretin family, glucagon-like peptide-1 (GLP-1)-receptor agonists and dipeptidyl peptidase-4 (DPP-4) inhibitors, are now available but at a higher cost than other drugs with many years in the market. Consequently, several authors have studied the cost-effectiveness of adding incretins to the treatment of patients with HbA_1c_ above those recommended by the International Diabetes Federation [13,14].

On account of the above mentioned data, the aim of the current study was to compare, from the perspective of the social security system, the simulated cost-effectiveness ratio of the treatment with saxagliptin (SAXA) *versu*s sulfonylureas (SU) as add-on therapy to MET (SAXA + MET *versus* SU + MET in a cohort of people with T2DM who did not attain HbA_1c_ treatment target values with metformin alone in a 20-year period.

## Methods

### Model structure and results

We used a stochastic simulation model especially designed to evaluate the impact of new therapies in people with T2DM (Cardiff Diabetes Model). A detailed description of its characteristics has been previously reported [15-17]. In brief, the model is a fixed-time-increment stochastic simulation based on the UKPDS 68 study [18]. The time increment is yearly and the model is designed to simulate a cohort of patients with T2DM (up to 10,000) over a 40-year time horizon. In general, the model runs twice; firstly for the ‘control’ group and secondly for the ‘treatment’ group.. Standard prediction from the model include the incidence of chronic microvascular (blindness, end-stage renal disease [ESRD] and neuropathy) and macrovascular (congestive heart failure, myocardial infarction, stroke and ischemic heart disease) complications, diabetes-specific mortality, and all-cause mortality. The model estimates costs and quality-adjusted life years (QALYs) associated with each treatment strategy, using only direct medical costs. Outputs include point and probabilistic estimates for cost-effectiveness.

### Population data and treatment strategy

The patient population included in the analysis has the demographic and associated cardiovascular risk factor (CVRF) (HbA_1c_ level, overweight/obesity and hypertension) profile of people with T2DM who need an add-on to MET treatment to achieve an HbA_1c_ treatment goal according to international guideline recommendations (Table 1). The cohort simulated has not a background of chronic complications or related events (Atrial fibrillation, peripheral vascular disease, ischemic heart disease, myocardial infarction, congestive heart failure, stroke, amputation, blindness and ESRD) [13]. The treatment strategies considered in the study were combination of SAXA + MET and SU + MET. Both treatment strategies were replaced by a rescue NPH insulin therapy when HbA_1c_ reached a pre-specified threshold value of 7.5%, as recommended by the national [19,20], as well as international guidelines for diabetes treatment [21].

**Table 1:** Inputs: Demographics characteristics

**Input**	Value*	**Reference**
**Baseline demographic characteristics**		
Current age (years, mean ± SD)	64 ± 10	[5,6,22-24]
Women (%)	47	[5,6,22-24]
Diabetes duration (years, mean ± SD)	10.5 ± 9.2	[5,6,22-24]
Height (m, mean ± SD)	1.52 ± 0.11	[5,6,22-24]
Smokers (%)	33	[5,6,22-24]
**Modifiable CVRF**		
HbA_1c_ (%, mean ± SD)	7.7 ± 1.8	[5,6,22-24]
Total cholesterol (mmol/l, mean ± SD)	5.2 ± 0.98	[5,6,22-24]
HDL-cholesterol (mmol/l, mean ± SD)	1.2 ± 0.4	[5,6,22-24]
SBP (mm Hg, mean ± SD)	131 ± 15	[5,6,22-24]
Weight (kg, mean ± SD)	70,8 ± 9,1	[5,6,22-24]

*Mean ± SD from a normal distribution in probabilistic sensitivity analysis.

### Treatment effectiveness and adverse effects

The model uses an effectiveness profile defined for each treatment (Table 2) that represents the impact on HbA_1c_ and body weight [14,25-29]. Additionally, adverse events (AE) associated to each treatment and their discontinuation probabilities are also defined for each effectiveness profile. By default, values for each treatment were taken from the single currently available head-to-head trial comparing saxagliptin plus metformin with sulfonylurea plus metformin [25]. Also, based on previously published data [26-30], in our simulation study we assumed that people treated with SAXA + MET should start this insulin rescue three years later than those receiving SU + MET.

**Table 2:** Inputs: Treatment effectiveness profiles

**Parameter**	**SAXA + MET**	**SU + MET**	**Reference**	**Insulin**	**Reference**
**HbA**_**1c**_					
Reduction in year 1 (%)	−0.57 (0.041)	−0.66 (0.041)	[23]	−1.0	Assumed
Delay in creep (years)	3.00	0.00	[25-28]	0.00	[25-28]
Body weight (kg)	−1,100 (0.017)	1,100 (0.018)	[23]	2,500	[24]
**Adverse Effect**					
**Hypoglycemic events**					
Number of symptomatic events	0.04 (0.02)	1.73 (0.08)	[23]	10.00	Assumed
Probability of seriousness	0.00	0.02	[23]	0.02	[24]
**Discontinuation probability**	0.00	0.00	Assumed	0.00	Assumed

Standard deviation (SD) in parentheses.

**Figure 1:**
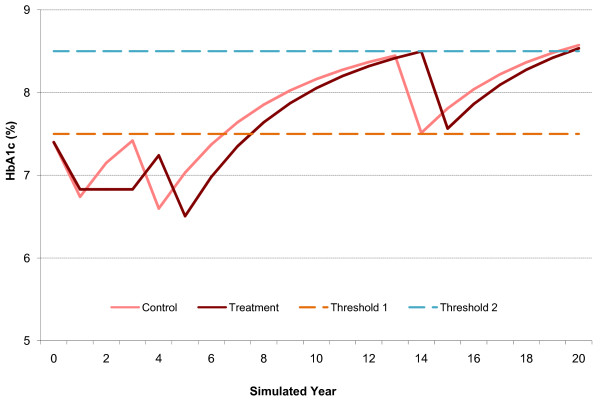
**HbA**_**1c **_**profile changes induced by the treatment tested along 20 years.**

Regarding the treatment effect upon HbA_1c_, it was assumed that in people with diabetes its values increase progressively and gradually [32]. To consider such progression, the model incorporates a “gradual increase delay” function, which allows the user to specify when HbA_1c_ starts its increment. For the current analysis, it was assumed that complete treatment effect occurs and is completely attained during the first year of treatment. Further, this value was considered as 0, assuming that the gradual HbA_1c_ increase as well as the change induced by SU + MET are immediate. In the case of SAXA + MET, such value was considered as 3. Figure 1 represents the effect of the different treatments mentioned in Table 2 upon HbA_1c_.

### Utilities

Since there are no specific utilities estimates from Argentina for diabetes and its complications, baseline utility was modelled using the mean EQ-5D values reported by the 2003 England Health Survey for people with diabetes and without major chronic complications, stratified by age group [31]. Utility decrements associated with complications were taken from the UKPDS study [32] with the exception of end-stage renal disease (ESRD) and blindness [33]. By default, subsequent events incurred the same utility decrement as in the initial event.

### Costs

Input data costs are those related to drug acquisition, AE and complication consequences. In the analysis, all costs are expressed in 2009 US dollars ($). Costs were initially calculated on Argentinean pesos and thereafter converted to US dollars at the official exchange rate of December 2009 ($1 = 3. 82 Argentinean pesos). All costs considered in the analysis are shown in Table 3.

**Table 3:** Inputs: Costs data

**Variable**		**Cost ($)**	**PSA**		
			**Distribution**	**Min**	**Max**
**Treatment**					
MET + SU (yearly)		217.99 *	-	-	-
MET * SAXA (yearly)		844.38 *	-	-	-
Insulin (yearly)		1,001.06*	-	-	-
**Adverse effect**					
Profound hypoglycemia		147.70**	Gamma	50	500
Symptomatic and nocturnal hypoglycemia		0.00 ^Δ^	-	-	-
**Macrovascular events**					
Ischemic heart disease	Fatal/nonfatal	1,204.2^†^	Gamma	500	4,500
	Maintenance	228.8‡	Gamma	100	500
Myocardial infarction	Fatal/nonfatal	1,548.1^†^	Gamma	500	4,500
	Maintenance	294.1‡	Gamma	100	500
Congestive heart failure	Fatal/nonfatal	719.9^†^	Gamma	500	4,500
	Maintenance	136.8‡	Gamma	100	500
Stroke	Fatal/nonfatal	942.4^†^	Gamma	500	4,500
	Maintenance	179.1‡	Gamma	100	500
**Microvascular events**					
Amputation	Fatal/nonfatal	789.3^†^	Gamma	500	4,500
	Maintenance	149.9‡	Gamma	100	500
Blindness	Fatal/nonfatal	390.4^†^	Gamma	200	2,000
	Maintenance	74.2‡	Gamma	50	500
ESRD	Fatal/nonfatal	13,759.2^†^	Gamma	10,000	30,000
	Maintenance	13,759.2^†^	Gamma	10,000	30,000

*Own data based on Alfabeta.net values (http://www.alfabeta.net); **Local Experts opinion; Δ assumption; † values paid by IOMA; ‡ estimated costs based on IOMA. PSA: Probabilistic sensitivity analysis.

For drug cost acquisition, unadjusted retail price was considered, in order to provide an approach for any kind of perspective by adjusting the scaling effect on total drug cost. The annual cost of drugs corresponded to the weighted cost of each drug, based on a combination dose of each one. The drug costs were obtained from Alfabeta.net, a private internet database which is the main source of drug pricing in the Argentine market. We considered the same price for saxagliptin and sitagliptin. Costs related to macrovascular and microvascular events were classified into fatal or non-fatal and applied to the year in which the event occurred. Maintenance costs for subjects who survived were applied in all subsequent years until the end of the simulation horizon or until the patient died. Costs of macrovascular and microvascular events were obtained from the reimbursement values paid in 2009 by a large local organization (Instituto de Obra Médico Asistencial [IOMA]) that belongs to the Argentinean social security health subsector at subnational level. Costs associated to microvascular events include costs caused by blindness/retinopathy, nephropathy, amputation and hypoglycemia. Maintenance costs are estimated based on expert opinion and values paid by IOMA. This includes physician visits, drugs and laboratory test. Indirect costs were not estimated.

### Discounting, Time Horizon, and Perspective

The analysis was taken from the perspective of Argentina social security health care system. The time horizon of the model was set to 20 years. Both costs and effects were discounted at a 3.5% annual rate.

### Sensitivity analysis

**Figure 2:**
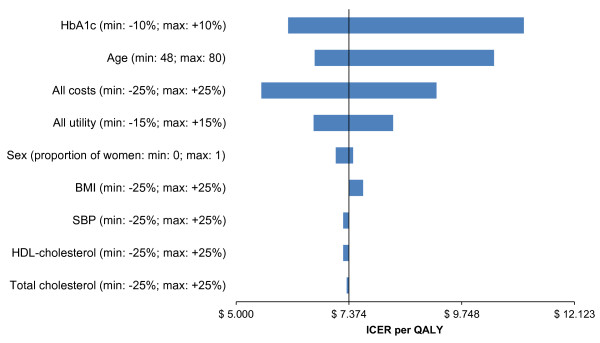
**Tornado diagram of the univariate sensitivity analysis showing the impact of individual input parameters on the ICER per QALY.** ICER: incremental cost effectiveness ratio. QALY: quality adjusted life year. Largest impact were associated to HbA1c, All utilities, Age and All costs changes.

We performed one-way sensitivity analyses to examine the robustness of results to variation in parameters and model assumptions. A one-way sensitivity analysis was performed for demographic (age and sex) and CVRF profile (HbA_1c_, systolic blood pressure [SBP], total cholesterol, HDL-cholesterol and body mass index [BMI]) variables, all costs and utilities (reported in detail in Figure 2). Additionally, probabilistic sensitivity analysis was performed using Monte Carlo simulation to evaluate the multivariate uncertainty in the model, *i.e.*, input parameters were varied simultaneously over specified ranges. Various probability distributions were chosen based on assumptions for each of these input parameters. A Normal, Gamma and Beta distribution was specified for demographics, costs and utility data, respectively. The Monte Carlo simulation drew values for each input parameter and calculated expected cost and effectiveness for each arm of the model. This process was repeated 10,000 times to give a range of all expected cost and effectiveness values and the results were illustrated as a cost-effectiveness acceptability curve.

## Results

### Base case

Over a 20-year time horizon, the model estimated that there will be less non fatal macrovascular and microvascular events in the group treated with SAXA + MET than in that treated with SU + MET (Table 4). Although only minor differences between groups were recorded in the number of events, those related to congestive heart failure and myocardial infarction were significantly lower in the SAXA + MET group. This fact could be ascribed to the different effect of the SAXA + MET and of the SU + MET combination upon body weight.

**Table 4:** **Metformin plus saxagliptin *vs. *metformin plus sulfonylurea: Events and costs**

	**MET + SU**		**MET + SAXA**		**Difference**	
	**Events**	**Cost**	**Events**	**Cost**	**Events**	**Cost**
**Macrovascular**						
IHD	99.2	193,759	99.0	193,285	−0.2	−474
MI	270.4	518,858	268.1	515,859	−2.3	−3,000
CHF	81.4	62,861	76.2	58,716	−5.2	−4,144
Stroke	90.6	111,876	89.9	111,084	−0.7	−792
**Microvascular**						
Blindness	60.5	36,879	60.6	36,503	0.2	−376
Nephropathy	13.0	420,397	12.9	410,562	−0.1	−9,834
Amputation	24.4	20,407	24.4	19,965	−0.1	−442
Hypoglycemia	1,179	128,719	1,032	108,438	−147	−20,281
Treatment	-	9,201,014	-	10,873,266	-	1,672,252
**Total**		10,694,769		12,327,677		1,632,909

*IHD*: ischemic heart disease; *MI*: myocardial infarction; *CHF*: congestive heart failure.

Similarly, the model predicted less number of deaths caused by macrovascular events in the group treated with SAXA + MET than in the SU + MET one (149.6 *vs.* 152.8).

Total costs were $12,327,677 for SAXA + MET and $10,694,769 for SU + MET. Table 4 shows that larger cost values corresponded to drug utilization, followed by myocardial infarction and nephropathy.

On the other hand, the number of QALYs per patient for SAXA + MET was larger than that of SU + MET (9.54 *vs.* 9.32) (Table 5). Also, the addition of SAXA resulted in a greater number of LYG per patient compared to SU (20.84 *vs.* 20.76). The QALY gain with SAXA + MET compared with SU + MET treatment was 0.22 per patient. There was only a small difference in LYG (0.08 LYG per patient).

**Table 5:** Cost-effectiveness results

**Cost-effectiveness**	**MET + SU**	**MET + SAXA**	**Difference**
Discounted costs	10,694,769 (10,694.8)	12,327,677 (12,327.7)	1,632,909 (1,632.9)
Discounted QALYs	9,322 (9.32)	9,544 (9.54)	221 (0.22)
Discounted LYG	20,765 (20.76)	20,845 (20.84)	80 (0.08)
Cost per QALY			7,374.2
Cost per LYG			20,490.3

The number in brackets corresponds to the average value per patient.

Considering that mean incremental cost was $1,632, the cost per QALY gained with SAXA + MET was $7,374 (Table 5).

### Sensitivity analysis

The consequences of modifying parameters applied in the sensitivity analysis are shown in Figure 2. While the largest variations were associated to HbA_1c_ values, age, costs and utility values, negligible impact were associated to HDL-cholesterol, BMI and Total cholesterol changes.

Anyhow, the analysis suggests that cost-effectiveness results remain robust to plausible variations of the main assumptions used in the model.

**Figure 3:**
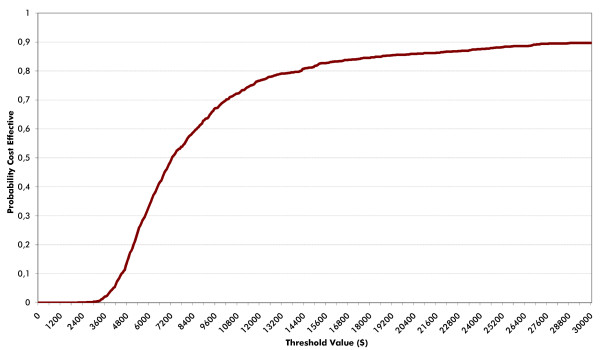
Cost-effectiveness acceptability curve.

The cost-effectiveness acceptability curve illustrates a probability of less than 58% that SAXA + MET is cost-effective compared with SU + MET (Figure 3), considering a willingness to pay of $7,626 (Gross Domestic Product *per capita* for Argentina) per QALY.

## Discussion

Based on the overall safety and effectivity of incretin-based therapies to achieve the treatment target value of HbA_1c_, earlier and more frequent prescription has been recommended for the treatment of people with T2DM [34]. Even when incretins have additional clinical value as repeatedly demonstrated [35], as any innovative pharmacotherapy its use increases the cost of treatment with the consequent negative impact upon health care budgets [36,37]. Therefore, different strategies have been used to demonstrate the relativity of such negative impact, based mainly on the verification of two key components: a) whether there is enough available evidence to support conclusions about the effectiveness of a given drug/intervention (adequacy of evidence), and b) whether that evidence implies about effectiveness (magnitude of benefit) [38,39]. This approach represents a marked change from earlier times when the doctor’s beliefs about the value of a drug/intervention were sufficient to establish medical necessity. Although different groups do not always reach the same conclusion about a particular drug/technology as well as on the suitability of the processes used to evaluate evidence, it is widely accepted that coverage determinations should be based on the results of carefully designed studies rather than on the expert opinion. The evidence-based evaluation is also used by the US Food and Drug Administration (FDA) for the approval process of a drug and by the Canadian and US task forces on preventive services [40,41]. In this context, our study analyzed the cost-effectiveness ratio of SAXA as add-on therapy to MET *versus* addition of SU in a cohort of people with T2DM who did not attain, in Argentina, treatment target values of HbA_1c_ with MET alone. The results indicate that the combination SAXA + MET produced a greater number of LYG and QALYs than that of SU + MET, with an incremental cost-effective ratio (ICER) equal to $ 7,374.

Since in Argentina there is not an accepted universal criterion to define a threshold for cost-effectiveness ratio, we used guidelines specifically intended for international comparisons, as proposed by the Commission on Macroeconomics and Health [42]. This criteria considered a strategy as "cost-effective" when the ICER was less than three times the gross domestic product (GDP) *per capita,* and as "very cost-effective" if the ICER was less than the GDP *per capita*[42,43]. Since in Argentina the GDP per person (current dollars) in 2009 was $7,626 [44], our SAXA + MET treatment strategy would be considered “very cost-effective”. Further, the sensitivity analyses showed that these results were robust to changes in input parameters.

Although in Latin America there are no similar studies to compare with, our results are similar to those reported by Schwarz *et al.* in a European population [14]. These authors evaluated the incremental cost-effectiveness of adding SITA to patients with HbA_1c_ > 6.5% while on MET in six European countries, using a discrete event simulation model which employed the UKPDS Outcomes Model risk equations for predicting risks of diabetes-related complications. They found that the discounted ICERs associated with the addition of SITA to MET, compared with adding a SU, the values ranging from €5,949 to €20,350 per QALY across countries. The sensitivity analyses showed that these results were robust to changes in input parameters, including clinical efficacy, costs and utility weights for both diabetes-related complications and hypoglycemia.

On the other hand, the NICE assessment group compared the rosiglitazone plus MET and a SU with sitagliptin plus MET treatment using the acquisition cost of the combined rosiglitazone/MET formulation for the analysis. They noted that the comparison of sitagliptin and rosiglitazone as well as that of vildagliptin and pioglitazone did not consider side effects associated with the use of thiazolidinediones. It was found that the sitagliptin intervention was the dominant option (*i.e.*, more effective and less costly than rosiglitazone), with or without considering complications at baseline [21].

Two simulation studies performed in Sweden and Germany using a similar approach to the one employed in our study were recently reported [45,46]. Although with differences in the monetary units used, in both cases the authors’ conclusions support our data, *i.e.*, over a patient's lifetime, the addition of SAXA to MET is associated with improvements in QALYs compared with SU in people with T2DM. Additionally, SAXA treatment was also a cost-effective alternative for this type of patients not well-controlled on MET alone.

Despite all the significant differences mentioned above, we must accept that the model used, and consequently the results/conclusions obtained, have some limitations, namely: 1) it only considered direct costs from a third party payer´s perspective; 2) it does not include nonmedical costs such as productivity lost and consequently it likely underestimates costs from a societal perspective; 3) the costs included in the analysis were obtained from a single institutional source (IOMA), no matter that IOMA is one of the most important institutions of social security system of Argentina and it is a reference for other institution. However, the update of the model results with additional sources of costs will improve its external validity; 4) data regarding utilities came from European population, which could be different for Argentinean one and thus it could have biased our results. In this regard we could argue that utility values were varied in the sensitivity analysis to show the impact on the outcomes of the model, and all the results of such sensitivity analysis variations confirmed those of our base case analysis. On the other hand, despite great efforts have been currently made to use the most accurate and up-to-date data sources to provide a realistic simulation of T2DM in Argentina, the model has a limitation shared by most modeling studies, namely, the uncertainty around projecting long-term outcomes based on clinical input data from a short-term study. This situation is conditioned by the absence of lifetime follow-up data from a well-designed clinical or epidemiological study. All these concerns suggest that the current results might be used with caution to avoid misleading conclusions rather than to deny their real value for decision makers and administrators.

## Conclusions

In brief, the data currently obtained using a stochastic simulation model designed to evaluate the impact of new therapies in people with T2DM [15-17] and local costs of drugs and diabetes-related events in a 20-year time horizon, strongly suggest that according to the criteria proposed by the Commission on Macroeconomics and Health, the use of the combination SAXA + MET in Argentina is very cost effective. We expect that these results will prompt our health care organizations to use these data, and also apply a similar procedure to the one currently described to take decisions on drug coverage. Implementation of such procedure would help to establish appropriate priorities to allocate economic resources based on objective evidence.

## Competing interests

The authors declare that JEC, JFE, JJG and LG have no competing interests, while EA and MW are employees of Bristol Myers Squibb.

## Authors' contributions

JJG conceived the study and coordinated the project. JEC, JFE, JJG and LG were responsible for literature review, real world-data acquisition, data analysis and manuscript writing. EA and MW cooperate in the literature search and access to as well as in the final revision of the manuscript. All authors read and approved the final version of the manuscript.
